# Supplementary material for: Unravelling the potential of nitric acid as a surface modifier for improving the hemocompatibility of metallocene polyethylene for blood contacting devices
Source: PeerJ. 2016 Jan 19;4:e1388. doi: 10.7717/peerj.1388 (PMC4727976; doi:10.7717/peerj.1388)
Supplement: Supplemental Information 1 — PT demonstrated an increase in their value for acid treated samples compared to the control. Mean PT of untreated sample was observed to be 19.23 s, whereas 30 and 60 min HNO3 exposed samples shown 19.86 s and 21.4 s, respectively indicating improved hemocompatibility. [file peerj-04-1388-s001.doc]

**Prothrombin Time (PT)**

| **Untreated Control** | **30 min HNO3** | **60 min HNO3** |
| --- | --- | --- |
| 19.3 | 19.6 | 20.8 |
| 19.5 | 20.1 | 21.4 |
| 18.9 | 19.9 | 22.0 |
