# Supplementary material for: Unravelling the potential of nitric acid as a surface modifier for improving the hemocompatibility of metallocene polyethylene for blood contacting devices
Source: PeerJ. 2016 Jan 19;4:e1388. doi: 10.7717/peerj.1388 (PMC4727976; doi:10.7717/peerj.1388)
Supplement: Supplemental Information 2 — Mean APTT was found to be 105.66 s, 113 s and 136.33 s for untreated, 30 min and 60 min acid treated mPE, respectively elucidating improved blood compatibility. [file peerj-04-1388-s002.docx]

**Activated Partial Thromboplastin Time (APPT)**

| **Control** | **30 min HNO3** | **60 min HNO3** |
| --- | --- | --- |
| 110 | 110 | 137 |
| 100 | 118 | 140 |
| 107 | 111 | 132 |
