# Supplementary material for: Unravelling the potential of nitric acid as a surface modifier for improving the hemocompatibility of metallocene polyethylene for blood contacting devices
Source: PeerJ. 2016 Jan 19;4:e1388. doi: 10.7717/peerj.1388 (PMC4727976; doi:10.7717/peerj.1388)
Supplement: Supplemental Information 3 — Mean absorbance seemed to decrease in the case of treated samples (0.02 and 0.007 for 30 min and 60 min HNO3-treated samples) compared with the untreated (0.05) mPE, indicating lesser damage incurred and interaction between the treated samples and RBC. [file peerj-04-1388-s003.docx]

**The Absorbance of Control and HNO_3_-treated Metallocene Polyethylene**

| **Positive Control** | **Control** | **30 min HNO_3_ Treated mPE** | **30 min HNO_3_ Treated mPE** |
| --- | --- | --- | --- |
| 0.66 | 0.06 | 0.029 | 0.008 |
| 0.69 | 0.04 | 0.021 | 0.008 |
| 0.61 | 0.06 | 0.031 | 0.007 |
