# Supplementary material for: Unravelling the potential of nitric acid as a surface modifier for improving the hemocompatibility of metallocene polyethylene for blood contacting devices
Source: PeerJ. 2016 Jan 19;4:e1388. doi: 10.7717/peerj.1388 (PMC4727976; doi:10.7717/peerj.1388)
Supplement: Supplemental Information 4 — The number of platelets adhered to a surface of treated polymers was found to be reduced to a great extent compared to the number of platelets which was found in the untreated sample. A maximum of 22 platelets was observed on the surface of the untreated samples, meanwhile the number of platelets decreased to a maximum of 15 platelets on 60 min treated samples. [file peerj-04-1388-s004.docx]

**Platelet Adhesion Assay of Untreated and HNO_3_-treated Metallocene Polyethylene**

| **Untreated** | **60 min HNO3 Treated mPE** |
| --- | --- |
| 22 | 13 |
| 25 | 15 |
| 21 | 17 |
