# Supplementary material for: Unravelling the potential of nitric acid as a surface modifier for improving the hemocompatibility of metallocene polyethylene for blood contacting devices
Source: PeerJ. 2016 Jan 19;4:e1388. doi: 10.7717/peerj.1388 (PMC4727976; doi:10.7717/peerj.1388)
Supplement: Supplemental Information 5 — The mean contact angle of the control was found to be 86.06°. This was found to be far greater in comparison to the acid treated samples. The mean contact angles of 30 and 60 min treated samples are 72.03°and 69.73°, respectively, dictating improved hydrophilicity to improve blood compatibility of mPE. [file peerj-04-1388-s005.docx]

| **Sample** | **Trial 1** | **Trail 2** | **Trail 3** |
| --- | --- | --- | --- |
| Untreated mPE | 84.9 | 87.2 | 86.1 |
| mPE treated with  HNO_3_ (30 min) | 72 | 74.1 | 70 |
| mPE treated with  HNO_3_ (60 min) | 68.2 | 71 | 70 |

**Contact Angle Measurement of the mPE Before and After HNO_3_ Treatment**
